# Watermelon Rind Mediated Biosynthesis of Bimetallic Selenium-Silver Nanoparticles: Characterization, Antimicrobial and Anticancer Activities

**DOI:** 10.3390/plants12183288

**Published:** 2023-09-17

**Authors:** Amr H. Hashem, Gharieb S. El-Sayyad, Abdulaziz A. Al-Askar, Samy A. Marey, Hamada AbdElgawad, Kamel A. Abd-Elsalam, Ebrahim Saied

**Affiliations:** 1 Botany and Microbiology Department, Faculty of Science, Al-Azhar University, Nasr City 11884, Cairo, Egypt; 2 Microbiology and Immunology Department, Faculty of Pharmacy, Ahram Canadian University, 6th of October City 12451, Giza, Egypt; adham_adham699@yahoo.com; 3 Microbiology and Immunology Department, Faculty of Pharmacy, Galala University, New Galala City 43511, Suez, Egypt; 4 Drug Microbiology Lab., Drug Radiation Research Department, National Center for Radiation Research and Technology (NCRRT), Egyptian Atomic Energy Authority (EAEA), Cairo 13759, Egypt; 5 Department of Botany and Microbiology, Faculty of Science, King Saud University, P.O. Box 2455, Riyadh 11451, Saudi Arabia; aalaskara@ksu.edu.sa (A.A.A.-A.); samarey@ksu.edu.sa (S.A.M.); 6 Integrated Molecular Plant Physiology Research (IMPRES), Department of Biology, University of Antwerp, 2020 Antwerp, Belgium; 7 Plant Pathology Research Institute, Agricultural Research Center, Giza 12619, Egypt

**Keywords:** watermelon rind, green biosynthesis, bimetallic nanoparticles, antimicrobial activity, anticancer activity

## Abstract

One of the most hazardous diseases that influences human health globally is microbial infection. Therefore, bimetallic nanoparticles have received much attention for controlling microbial infections in the current decade. In the present study, bimetallic selenium–silver nanoparticles (Se-Ag NPs) were effectively biosynthesized using watermelon rind WR extract through the green technique for the first time. UV-visible spectroscopy, transmission electron microscopy (TEM), and energy dispersive X-ray spectroscopy (EDX) methods were used to characterize the produced NPs. The results indicated that the bimetallic Se-Ag NPs had synergistic antimicrobial activity at low concentrations, which helped to reduce the toxicity of Ag NPs after the bimetallic Se-Ag NPs preparation and increase their great potential. Se-Ag NPs with sizes ranging from 18.3 nm to 49.6 nm were detected by TEM. Se-Ag NP surfaces were uniformly visible in the SEM picture. The cytotoxicity of bimetallic Se-Ag NPs was assessed against the Wi38 normal cell line to check their safety, where the IC_50_ was 168.42 µg/mL. The results showed that bimetallic Se-Ag NPs had antibacterial action against *Candida albicans*, *Escherichia coli*, *Pseudomonas aeruginosa*, *Klebsiella oxytoca*, *Bacillus subtilis*, and *Staphylococcus aureus* with a minimum inhibitory concentration (MIC) of 12.5 to 50 µg/mL. Additionally, bimetallic Se-Ag NPs had promising anticancer activity toward the MCF7 cancerous cell line, where the IC_50_ was 21.6 µg/mL. In conclusion, bimetallic Se-Ag NPs were biosynthesized for the first time using WR extract, which had strong antibacterial, antifungal and anticancer properties.

## 1. Introduction

In the last two decades, antimicrobial resistance has been considered one of the most serious problems affecting human health around the world [1]. Antimicrobial resistance refers to the ability of a microorganism to resist the effects of various antimicrobial agents. In this particular form of resistance, microorganisms have the ability to withstand the effects of medication that was previously effective against them [2]. Multidrug resistance (MDR) refers to the phenomenon where resistance develops against multiple drugs. Microbes are able to show a range of resistance mechanisms, encompassing innate resistance displayed by certain microbes against specific antimicrobials, genetic mutations, and acquired resistance obtained from other species [3]. The abuse of antibiotics led to the development of many multidrug resistant bacteria [4]. The widespread dispersion of Gram-positive (*Bacillus cereus*, *Staphylococcus aureus*), Gram-negative (*Escherichia coli*, *Klebsiella pneumonia*), and nosocomial infections in the natural environment are two of the most significant causes of disease in humans, especially nosocomial infections [5]. Given that pathogenic bacteria are often resistant to typical bactericidal agents prevalent in the environment, there is a need to develop innovative, reasonably priced, and environmentally friendly bactericidal agents. These new bactericidal potentials for nanoparticles present themselves [6,7].

The rapid advancement of nanotechnology has shown how deeply it depends on modern science and technology. Additionally, it integrates multidisciplinary fields including materials science, chemistry, physics, biology, and medicine [8]. As a result, the synthesis of nanoparticles with unusual characteristics has drawn considerable interest for a variety of applications. Nanoparticles offer better physical, chemical, and biological characteristics than larger particles made from the same precursor due to their smaller size range [9]. Numerous scientific and technological research fields have shown a great deal of interest recently in nanoparticles with superior bactericidal activity and low cytotoxicity. The biosynthetic process is less effective when agricultural products with a high economic value are used to make nanoparticles (NPs). Utilizing agricultural waste is an environmentally friendly way to create NPs that have the potential to be used in biological applications [10]. The green synthesis of metallic NPs uses a variety of agrowastes because they are rich in biomolecules that act as bioreductant agents, such as phenolics, alkaloids, flavonoids, proteins, carbohydrates, and amino acids. These include rice bran, corn cob, coconut coir, wheat bran, fruit seeds, palm oil mill effluent, groundnut bran, and peels, as well as biological sources and enzymes [11,12]. One of the fruits that is most often consumed worldwide is the watermelon (*Citrullus lanatus*) [13]. Watermelon’s red flesh is edible, but the rind is discarded because it has no use in commerce. However, cellulose, citrulline, pectin, proteins, and carotenoids are only a few of the bioactive substances that are abundant in watermelon rind [14]. The rind of the watermelon includes soluble carbohydrates (45–65%), carotenoids, alkaloids, saponin, and phytates. WMSs are an excellent source of protein (15–50%) such as albumin, globulin, prolamin, and glutelin. WMSs are also a good source of vitamin B-complex (B1, B2, B3, B6 and B12), polyunsaturated fatty acids, essential and non-essential amino acids as well as phenolic compounds. Moreover. Watermelon by-products also present therapeutic properties including anti-diabetic, antioxidant, antihypertensive, anti-inflammatory, antiulcer, antitumor, hypocholesterolemic, hepato-, nephron- and neuro-protective effects and antibacterial properties [15,16]. Silver (Ag), selenium (Se), and their bimetallic alloy NPs must become more important among the metal NPs by pioneering optical, physical, chemical, catalytic, photothermal, and electrical contributions for a variety of applications. Antibacterial, anticoagulant, antioxidant, larvicidal, anticancer, and thrombolytic are only a few of the significant uses for these NPs [17,18]. The treatment efficacy increases and the negative effects decrease when the particle size is decreased. The FDA and other health organizations have approved many pharmaceutical companies’ nano-formulation-based medications during the past ten years [19]. Numerous cases of selenium being used to treat cancer have already been documented, but the mechanism underlying this activity is still unclear [20]. The biological production of silver and selenium NPs had previously been shown in the literature [21,22,23,24]. In comparison to organic and inorganic Se-compounds like Se (IV) and Se (VI), nano-selenium exhibits higher biological activity, better bioavailability, and lower toxicity. This study aims to biosynthesize and characterize bimetallic nanoparticles (selenium–silver NPs) using watermelon rind for the first time, and also to assess their antimicrobial as well as anticancer activities.

## 2. Results and Discussion

### 2.1. Biosynthesis of Bimetallic Se-Ag NPs Using WR

The process of NPs preparation from plants is straightforward; at the typical room temperature, a plant extract may manufacture metal nanoparticles in a matter of minutes to several hours. This technique has received a lot of interest in the past 10 years because Ag and Se nanoparticles are more stable and safer at low concentrations than other bimetallic nanoparticles. The outcomes of producing nanoparticles from different plant organ extracts varied [25]. Fruit peel wastes are an excellent source of phytochemicals, which play a key role in the creation of nanocomposite materials. Different plant organ extracts had different outcomes when used to make nanoparticles. In our study, bimetallic nanoparticles were produced within 15 min of incubation, and visual inspection revealed a continuously increasing rate of synthesis. The solution’s color changed from clear to a deep reddish brown after the synthesis of nanoparticles (bimetallic Se-Ag NPs). The NPs’ stability in colloidal form was also helped by this capping agent (WR extract). In addition to reducing the ions needed to prepare the NPs, the presence of biomolecules in the extract even at low concentrations also prevents the bulk of the generated nanoparticles from aggregating. Similar studies showed that a considerably higher extract ratio is necessary for the creation of symmetrical nanoparticles [26,27,28]. The colloidal solution of nanoparticles exhibits color variation based on the size and shape of the particles due to surface plasmon resonance. The main phytochemicals present in plant peels that contribute to bio-reduction and NP production include sugars, ketones, terpenoids, carboxylic acids, aldehydes, flavones and amides [29]. More tannin was found in peels, leaves, and flowers than in seeds, whereas peels, seeds, and flowers had more saponins than leaves [30]. Different functional groups found in flavonoids have an improved capacity to reduce metal ions [31]. The first step in the creation of various NPs was the dissolution of AgNO_3_ and Na_2_SeO_3_, which produced hydrated cations (Ag^+^ and Na^+^) and anions (SeO_3_^−2^ and NO_3_^−^).

Additionally, the higher total phenolic contents of the peel extracts from pomegranate and watermelon help to reduce silver ions to Ag NPs [32,33]. According to Mittal et al. [34], who successfully used quercetin and gallic acid to induce the production of bimetallic (Ag-Se) nanoparticles. Au-Ag BNPs were successfully synthesized swiftly and persuasively using an aqueous *Plumbago zeylanica* root extract [35]. According to Godipurge et al. [36], under microwave irradiation, bioactive chemicals from the aerial parts of *R. hypocrateriform* is were successfully employed to generate Au-Ag alloys. According to Mostafa et al. [37], the extracts from orange and pomegranate peels have higher total phenolic contents, which support the reduction of silver ions to Ag NPs. Pomegranate peels and nanochitosan were used by Salem et al. [38] to biosynthesize selenium nanoparticles. Fardsadegh and Jafarizadeh-Malmiri [39] employed *aloe vera* leaf extract to aid in the green manufacturing of selenium nanoparticles.

### 2.2. Characterization of the Biosynthesized Se-Ag BNPs

#### 2.2.1. UV-Vis

**Figure 1:**
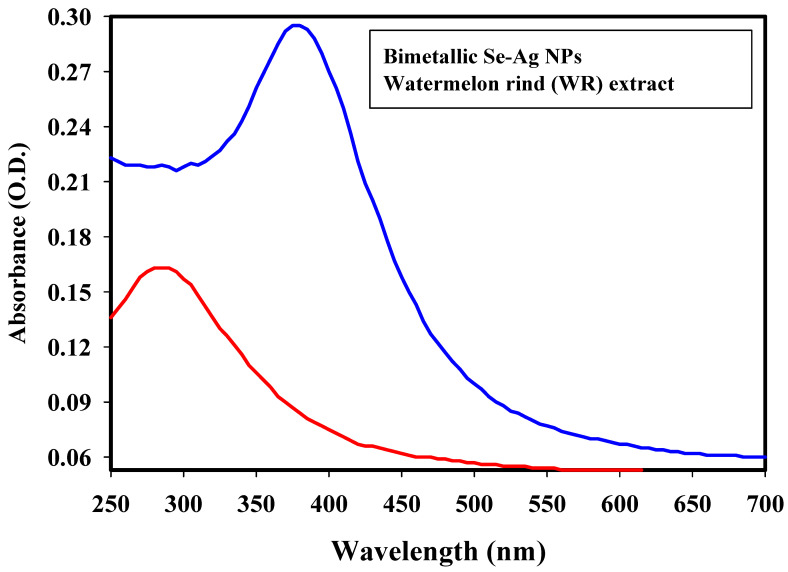
UV-vis absorption spectra of bimetallic Se-Ag NPs and WR.

WR filtrate was tested for its capabilities regarding bimetallic Se-Ag NPs biosynthesis. The color of the WR seemed yellow, which shifted to a deep reddish brown concerning the biosynthesis of bimetallic Se-Ag NPs. The produced color was assigned to the stimulation of biogenic Se-Ag NPs’ surface plasmon resonance and provided a specific wavelength of their appearance [40]. The capacity of WR filtrate to produce bimetallic Se-Ag NPs was evaluated. The biogenic Se-Ag NPs surface plasmon resonance was attributed to the bimetallic Se-Ag NPs, which also gave a reliable spectroscopic indication of their presence [41]. The experimental peak was visible in the UV-visible spectrum (Figure 1) due to the O.D. (0.295; diluted 30 times), and the spectrum was found at 380 nm. The intensity of the deep reddish brown constructed was fitting for the power of the prepared WR to biosynthesize bimetallic Se-Ag NPs.

In contrast, Mirzaei et al. [42] showed that the synthesis of ZnSe NPs was visually confirmed by the change of color solution and the UV–vis spectrum revealed two absorption peaks in the 250 nm and 360 nm regions associated with the formation of ZnSe NPs by using of the seaweed extract. According to Olawale et al. [21], the surface plasmon resonance bands at 433 nm confirmed the synthesis of the Ag and Se NPs by using an aqueous *Ocimum tenuiflorum* inflorescence extract and the peak of the extract was detected at 285 nm. Surface plasmon resonance is often influenced by the size, shape, morphology, structure, and dielectric properties of any nanoparticles created [43,44].

#### 2.2.2. TEM and DLS Analysis

**Figure 2:**
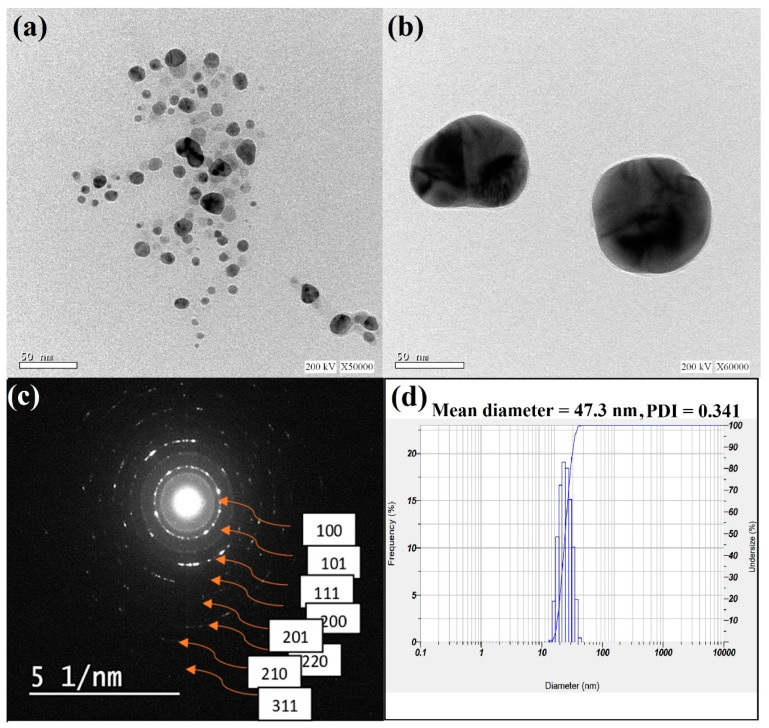
(**a**,**b**) TEM images of bimetallic Se-Ag NPs at different magnifications; (**c**) SAED pattern of Se-Ag BNPs.; (**d**) nanoparticle size distribution histograms for Se-Ag BNPs.

The produced bimetallic Se-Ag NPs underwent TEM examination to determine the average particle size and disclose their appearance. Additionally, comparisons between TEM and DLS data were made. The produced bimetallic Se-Ag NPs had spherical and oval shapes, which were visible in TEM images. The scale of Se-Ag NPs is shown in Figure 2a,b, and the average size was found to be 24.5 nm, ranging from 18.3 nm to 49.6 nm. SAED is a crystallographic experimental technique that can be performed within a transmission electron microscope (TEM). Obviously, the image is a series of spots, each spot corresponding to a satisfied diffraction condition of the sample’s crystal structure. If the samples are tilted, the same crystal will stay under illumination, but different diffraction conditions will be activated, and different diffraction spots will appear or disappear. The spotted circles on the pattern correspond to the pure phase of the bimetallic Se-Ag NPs structure. The pattern is indexed to (100), (101), (111), (200), (201), (220), (210) and (311) of the fcc of bimetallic Se-Ag NPs, respectively. These patterns are in accordance with the results. The pattern also showed that a powder is polycrystalline in nature (Figure 2c). Castro-Longoria et al. [45] generated silver, gold, and silver-gold bimetallic NPs using the extract of a filamentous fungus, and it was found that the NPs were predominantly spherical with average diameters of 11.0 nm for silver and 32.0 nm for gold, respectively. The anisotropic form and partial stability of the NPs in our work are caused by using of reducing and capping agents (WR extract). Finally, the outcomes were connected to the most recent publications [40]. Bimetallic Se-Ag NPs were subjected to DLS analysis in order to assess the particle size distribution and determine the average particle size, which was determined to be 47.3 nm (Figure 2d). Since TEM analysis determines the actual particle size of the material without the solvent layer while DLS analysis measures the hydrodynamic radius of NPs bound by water molecules (solvent), resulting in larger particle sizes of the capped NPs, it is typical for DLS size measurements to have higher values than TEM measurements [46]. According to the current DLS data, all of the synthesized NPs were evenly dispersed, with a PDI of 0.341.

#### 2.2.3. SEM and EDX Mapping Analysis

**Figure 3:**
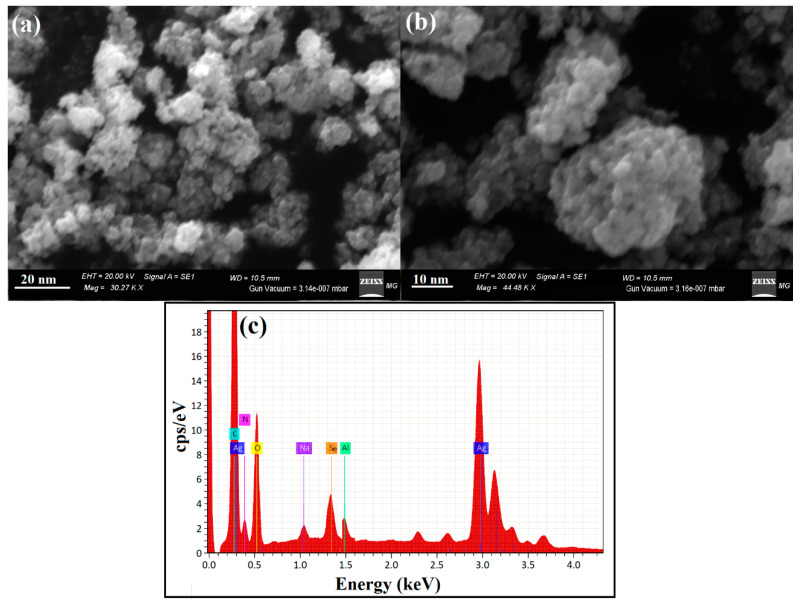
SEM image at different magnifications (**a**,**b**) and corresponding EDAX spectra (**c**) of the synthesized bimetallic Se-Ag NPs.

SEM micrographs were used to determine the diameter of the particles (∼100) and to characterize the size distribution of the particles in each batch. The surface morphology and elemental analysis of the bimetallic Se-Ag NPs that have been synthesized are depicted in Figure 3a–c. The topography structure of synthesis bimetallic Se-Ag NPs at low magnification exhibited particles that aggregated as spheres and formed a tortuous surface. EDX spectroscopy was used to confirm the generated samples chemically and evaluate their elemental structure and composition [47,48]. Se and Ag were uniformly distributed as the bimetallic Se-Ag NPs, which appear as bright particles over the WR extract (Figure 3a,b). As shown in Figure 3c, the purity and fundamental structure of the synthesized bimetallic Se-Ag NPs were determined using an EDX examination. On the other hand, the EDX chart affirmed the presence of C, Na, Al, N, and O atoms due to the WR plant extract.

#### 2.2.4. SEM/EDX Elemental Mapping Analysis of Bimetallic Se-Ag NPs

**Figure 4:**
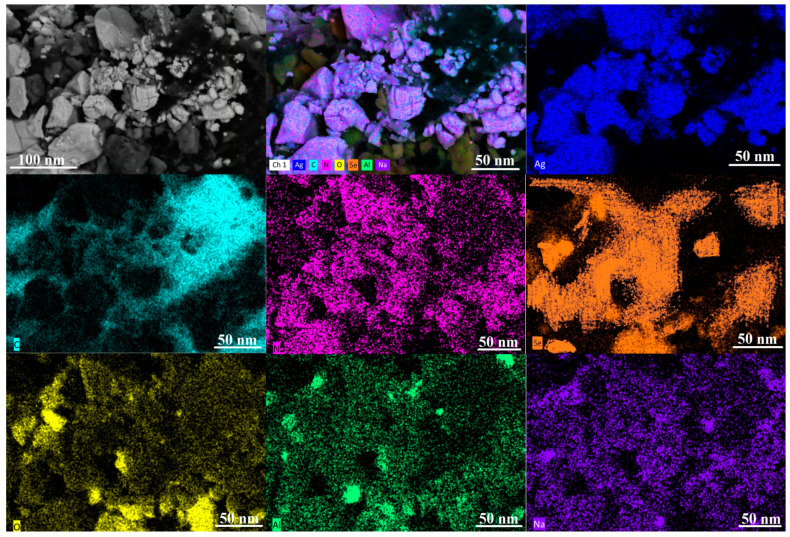
SEM/EDX Elemental mapping analysis of bimetallic Se-Ag NPs.

The elemental mappings of bimetallic Se-Ag NPs are displayed in Figure 4. All images are identified as Ag, Se, O, N, Al, Na, and C for bimetallic Se-Ag NPs. From this figure, it is obvious that Se-Ag NPs are similar in terms of the appearance of Ag and Se atoms, where both are homogenously distributed. Also, O, N, Al, Na, and C atoms were present, where (O, N, and C) for the plant extract [49] used for bimetallic synthesis, and other elements (Al, and Na) were impurities during the imaging process.

#### 2.2.5. XRD Analysis and FTIR Determination

**Figure 5:**
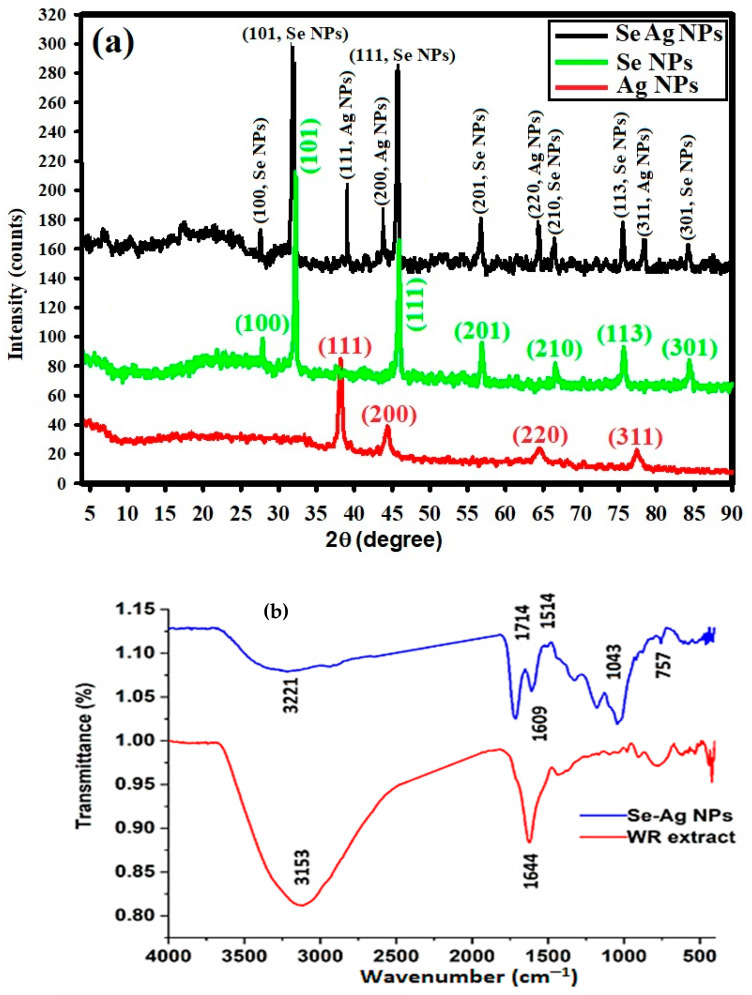
(**a**) XRD patterns of bimetallic Se-Ag NPs and WR; (**b**) FTIR analysis of bimetallic Se-Ag NPs and WR extract.

XRD analysis was used to examine the bimetallic Se-Ag NPs’ crystal structure and phase as well as the precursor’s crystal arrangements, which were compared with our synthesized Ag NPs and Se NPs alone. Figure 5a shows the XRD pattern of the synthetic bimetallic Se-Ag NPs. The production of the nanocomplex (Se-Ag NPs) was verified by XRD findings. XRD diffraction peaks of Ag NPs are shown in Figure 5a, including peaks at 2θ = 38.48°, 44.09°, 63.90°, and 78.31° that correspond to (111), (200), (220), and (311) Bragg’s reflections, respectively, and are matched with a reference card JCPDS-ICDD card 04-0783 [50]. XRD analysis was used to examine the bimetallic Se-Ag NPs’ crystal structure and phase. Additionally, Figure 5a shows the XRD diffraction peaks of selenium nanoparticles, including peaks at 2 θ of the 27.92° (100), 31.8° (101), 47.84° (111), 57.44° (201), 65.64° (210), 76.3° (113), and 84.0° (301) conventional selenium nanoparticle crystal planes, respectively [51], demonstrating the crystal nature of the synthesized bimetallic Se-Ag NPs with polycrystalline configuration.

Finally, the Debye–Scherrer equation was used to determine that the mean bimetallic Se-Ag NPs crystallite size was 23.84 nm. The generated NPs were highly crystalline for improved application, according to the XRD data [52]. 

The bimetallic Se-Ag NPs and WR extract were characterized by FTIR spectroscopy. Figure 5b shows the FT-IR spectra of the bimetallic Se-Ag NPs and WR extract, which are analyzed in the range between 400 cm^−1^ to 4000 cm^−1^. The spectra of the bimetallic Se-Ag NPs exhibit varied bands at 413, 427, 477, 573, 608, 757, 919, 1043, 1177, 1323, 1514, 1609, 1714 and 3221 cm^−1^. In FTIR analysis, the −OH functional group is seen to link to wavenumbers at 3221 and 3153 cm^−1^ (Figure 5b). The band at 1714 cm^−1^ was due to carbonyl group C=O indicating the presence of ketones, aldehydes, and carboxylic acids. The short moderate peak at 1609 cm^−1^ shows the existence of unsaturated combinations (alkenes). The C−O stretching mode for the amide group may be responsible for the band at 1043 cm^−1^ [53]. Extracts from many plants have been studied for the production of various kinds of NPs [54,55]. The −CH_3_O deformation band is at 1514 cm^−1^. Finally, a noted peak at 757 cm^−1^ for −CCH and −COH bending, from Figure 5b, can show that the WR spectra are identical to that declared in the literature review. The shifts in the peaks indicate that the organic components in the extract of WR successfully promoted the synthesis of bimetallic Se-Ag NPs during the reduction process and may aid in preventing the bimetallic Se-Ag NPs from aggregating and so maintaining their long-term stability [32,33].

### 2.3. Antimicrobial Activity

**Figure 6:**
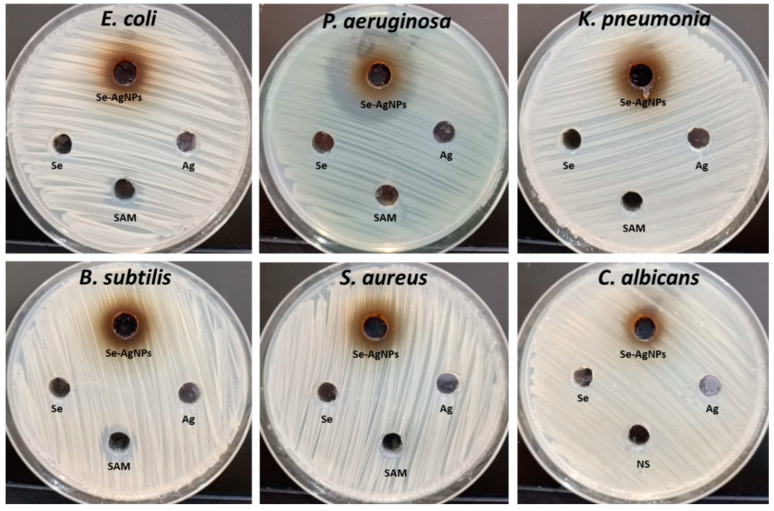
Antimicrobial activity of bimetallic Se-Ag NPs, sodium selenite (Se), silver nitrate (Ag), Ampicillin-Sulbactam (SAM) and Nystatin (NS) toward *E. coli*, *P. aeruginosa*, *K. pneumonia*, *B. subtilis*, *S. aureus* and *C. albicans*.

Bimetallic nanoparticles have received much attention in the last period due to the fact that monometallic nanoparticles have fewer efficacies on multidrug resistant microbes [56]. Therefore, in this study, bimetallic Se-Ag NPs were evaluated for antimicrobial activity against bacterial and fungal strains. The agar-well diffusion technique was used to evaluate the antibacterial activity of bimetallic Se-Ag NPs, as shown in Figure 6. The results revealed that bimetallic Se-Ag NPs exhibited antimicrobial activity toward Gram-positive, Gram-negative and unicellular fungi. Furthermore, *K. oxytoca* was the most sensitive among other microbial strains, where the inhibition zone was 20.00 ± 1.00 mm at a concentration of 100 µg/mL of bimetallic Se-Ag NPs as shown in Table 1. Moreover, bimetallic Se-Ag NPs showed antibacterial activity against *E. coli*, *P. aeruginosa*, *B. subtilis* and *S. aureus* where inhibition zones were 14.33 ± 0.58, 18.87 ± 0.76, 16.17 ± 0.76 and 17.17 ± 1.26 mm, respectively. Also, bimetallic Se-Ag NPs exhibited weak antifungal activity toward *C. albicans* where the inhibition zone was 12.27 ± 1.42 mm.

Moreover, different concentrations of bimetallic Se-Ag NPs were evaluated as antimicrobials to detect the minimum inhibitory concentration (MIC). Results illustrated that MICs toward *P. aeruginosa*; *K. oxytoca* and *S. aureus* were 12.5 µg/mL for each one. Moreover, the MIC of Se-Ag NPs against *B. subtilis* was 25 µg/mL. On the other hand, both *E. coli* and *C. albicans* were the least sensitive among the other tested microbial strains, where the MIC was 50 µg/mL for each one. Mostafa, El-Sayyad [40] used *Orobanche aegyptiaca* extract for the biosynthesis of bimetallic Ag-Se NPs where it showed promising antimicrobial activity against *S. aureus*, *E. coli*, *P. aeruginosa*, and *C. albicans.* Also, bimatellic Ag-Se nanoparticles were synthesized using quercetin and gallic acid, where exhibited outstanding antimicrobial and antitumor activities [57]. Khalil, Hashem [58] succeeded in preparing a bimetallic hydrogel based on silver and copper, and results revealed that this hydrogel exhibited promising antibacterial activity toward *Escherichia coli*, *Pseudomonas aeruginosa*, *Streptococcus mutans*, *Staphylococcus aureus*, *Bacillus subtilis*, and *Candida albicans.* Furthermore, Hashem and El-Sayyad [59] reported that the biosynthesized Ag-ZnO NPs had potential antibacterial activity toward *E. coli*, *P. aeruginosa*, *B. subtilis*, *S. aureus*, and *E. faecalis;* also showed antifungal activity against *C. albicans*, *Cryptococcus neoformans*, *Aspergillus fumigatus*, and *A. brasiliensis.* Also, Padilla-Cruz, Garza-Cervantes [60] found that bimetallic Ag-Fe had higher antimicrobial efficacy toward yeast, Gram-negative and Gram-positive multidrug resistant bacteria than monometallic nanoparticles. Furthermore, plant extracts have been frequently used for the green biosynthesis of bimetallic nanoparticles in the last decade [61,62]. Furthermore, *Moringa oleifera* was used for the biosynthesis of Se-Cu bimetallic nanoparticles, where it exhibited high antimicrobial activity toward pathogenic bacteria compared to ampicillin [61]. Also, the extract of *Terminalia chebula* was used for the biosynthesis of silver-palladium, which had antimicrobial activity against both Gram-positive and Gram-negative bacteria [62].

Bimetallic NPs composed of two different metals can exhibit synergistic effects, where the combined action of the metals enhances the antibacterial activity. The interaction between the metals can result in increased ROS generation, improved metal ion release, and enhanced physical damage to bacterial cells [63]. Bimetallic NPs can physically damage bacterial cells; the small size and high surface area of NPs allow them to interact with the bacterial cell membrane, as well as interaction between positively charged NPs and negatively charged bacterial cell membranes, leading to disruption and leakage of intracellular components, then inhibition of bacterial growth and cell death [56,64]. Also, ions cause disturbances in hemostasis where they bind to the peptidoglycan layer’s SH groups, causing the cell wall to break down [65]. Moreover, metallic NPs produce ROS, which can cause oxidative stress in bacterial cells by damaging proteins, lipids, and DNA. The accumulation of ROS can disrupt cellular processes and lead to bacterial cell death [66].

### 2.4. Cytotoxicity and Anticancer Activity

**Figure 7:**
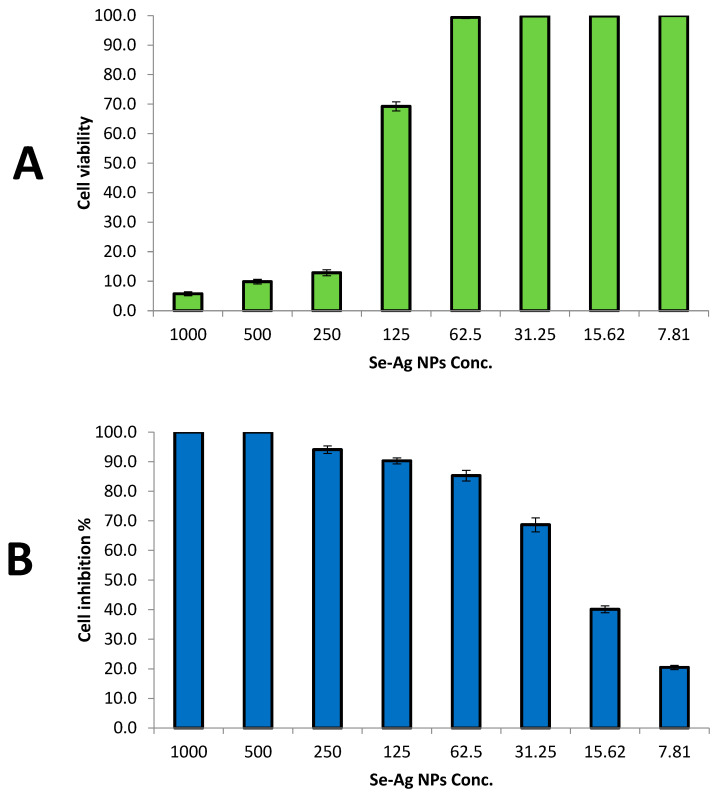
Cytotoxicity of Se-Ag NPs toward Wi38 normal cell line (**A**) and MCF7 cancerous cell line (**B**).

Evaluation of the cytotoxicity of new materials toward normal human cell lines is considered the first step to checking their safety in order for them to be used later by humans [67]. In the current study, the Wi38 normal cell line was used to evaluate the cytotoxicity of bimetallic Se-Ag NPs at different concentrations, as illustrated in Figure 7. Results revealed that low concentrations of bimetallic Se-Ag NPs at 7.81, 15.62 and 31.25 µg/mL have no cytotoxicity toward the Wi38 cell line (Figure 7A). On the other hand, high concentrations at 250, 500 and 1000 µg/mL have high cytotoxicity toward the Wi38 cell line, where cell inhibitions were 87.1, 90.2 and 94.3%, respectively. Furthermore, the IC50 was calculated at 168.42 µg/mL, which confirms bimetallic Se-Ag NPs are safe to use due to the fact that IC_50_ ≥ 90 μg/mL, the compound or material is classified as non-toxic [68].

Anticancer activity of bimetallic Se-Ag NPs was assessed using the MCF7 cancerous cell line, as shown in Figure 7B. Results illustrated that bimetallic Se-Ag NPs exhibited potential anticancer activity, where the IC_50_ was 21.6 µg/mL. Moreover, different safe concentrations of bimetallic Se-Ag NPs had potential anticancer activity in cell inhibition at 90.3, 85.3, 68.7, 40.2 and 20.5% at 125, 62.5, 31.25, 15.62 and 7.81 µg/mL, respectively. Herein, the biosynthesized bimetallic Se-Ag NPs showed high efficacy toward cancer cell line at safe concentrations, which can be used as safe anticancer agents after in vivo studies. Previous studies reported that bimetallic nanoparticles have anticancer activities against different cancerous cell lines [69]. Hashem and El-Sayyad [59] reported that the biosynthesized bimetallic Ag-ZnO NPs using pomegranate peel extract sowed promising anticancer activity toward CaCO2 and MCF7, where IC50 was 52.4 and 104.9 µg/mL, respectively, and where these concentrations were safe according to cytotoxicity results toward the vero normal cell line (IC50 = 155.1 µg/mL). Also, Elsayed, Alomari [70] confirmed that Ag-ZnO, synthesized by the laser ablation technique, can be used for cancer therapy, where it exhibited anticancer activity against HCT-116 and HELA cancerous cell lines. Moreover, Katifelis, Mukha [71] reported that the synthesized Ag-Au NPs showed antitumor and antimetastatic activities in vivo. Furthermore, bimetallic Au-Ag NPs appeared to have antiproliferative effects in HeLa (cervical cancer) and DU 145 (prostate cancer) cells [72]. Additionally, Mittal, Kumar [57] synthesized bimetallic Se-Ag NPs chemically using quercetin and gallic acid, where they showed anticancer activity toward Dalton lymphoma cells and an inhibition percentage of 80% at 50 µg/mL.

Metal polymer nanocomposite is thought to work as an anticancer agent by causing the creation of reactive oxygen species (ROS) as a result of oxidative stress on cancer cells. ROS can induce oxidative stress and cause damage to cancer cells by targeting cellular components like proteins, lipids, and DNA (DNA strand breaks, base modifications, and DNA cross-linking). The accumulation of ROS can lead to cell death through apoptosis or necrosis [73]. In addition to this, it is possible that it is caused by the electrochemical interaction of certain negative charges on the surface of cancer cells with the positive charges of metal NPs in the environment [74]. Furthermore, bimetallic NPs can disrupt the mitochondrial membrane potential, impair ATP production, and induce mitochondrial-mediated apoptosis, triggering cancer cell death [75]. Moreover, NPs can interfere with signaling pathways involved in angiogenesis, such as VEGF (vascular endothelial growth factor), thereby preventing the blood supply to tumors and inhibiting their growth [76].

## 3. Materials and Methods

### 3.1. Materials

The principal substances utilized in the synthetic process include AgNO_3_, Na_2_SeO_3_, and other reagents at the analytical standard grade from Sigma-Aldrich. Nutrient agar, nutrient broth and malt extract agar media were purchased from Hi Media. Without any additional purification, all reagents were used as supplied. In each experiment, deionized water was utilized.

### 3.2. Preparation of Watermelon Rind Extract

The watermelon rind [49] was collected from local markets in Giza City, Egypt. Watermelon rind extraction was carried out according to the method used by Ali, Hasanin [77].

### 3.3. Green Biosynthesis of Se-Ag BNPs

Se-Ag NPs were produced using a straightforward procedure. Solutions of Na_2_SeO_3_ and AgNO_3_ (2 mM each) were first made. The reaction mixture was as follows: Following the addition of 20 mL of Na_2_SeO_3_, 20 mL of AgNO_3_, and 60 mL of plant extract, the solution was vigorously stirred at 60 °C. Then, 1N NOaH was added to change the pH. The finished mixture was magnetically agitated for two hours at 60 °C during the procedure, and a change in color was eventually noticed, indicating the creation of Se-Ag NPs at pH 8.0 [39]. Centrifugation was used to separate the precipitate, which was then washed three times with deionized water and dried in an oven at 80 °C overnight [78].

### 3.4. Characterization of Se-Ag NPs BNPs

UV-Vis spectroscopy (JENWAY-6305, Staffordshire, UK) was used to initially characterize the phyto-synthesized Se-Ag NPs. To find the maximal surface plasmon resonance, 2 mL of the produced solution was added to the cuvette, and the spectra were measured between 200 and 800 nm [79]. An effective method for learning more about the crystallinity or amorphous characteristics of Se-Ag NPs produced by plants is X-ray diffraction. The XRD spectra were examined using X’ Pert Pro (Philips, Eindhoven, The Netherlands) at 2θ values between 10° and 80°. XRD analysis was used to compute the average crystal size using the Debye–Scherrer equation. Using a JASCO FT-IR 3600, the KBr Pellet technique, and a wavenumber range of 400 to 4000 cm^−1^, the Fourier transform infrared (FT-IR) spectra of phyto-synthesized Se-Ag NPs were detected. To study the size, shape, and aggregation of phyto-synthesized Se-Ag NPs, selected area electron diffraction (SAED) and transmission electron microscopy (JEM-1230, Akishima, Tokyo, Japan) were both utilized. Using a scanning electron microscope coupled to an energy dispersive X-ray device (SEM-EDX) (SEM, ZEISS, EVO-MA10, Germany) (EDX, Bruker, Mannheim, Germany), it was possible to determine the elemental compositions of the produced bimetallic Se-Ag NPs. The final analysis was performed using a zeta sizer (Nano-ZS, Malvern, UK) and dynamic light scattering (DLS) to assess the size distribution and polydispersity index (PDI) of Se-Ag NPs generated by using the green approach.

### 3.5. Antimicrobial Activity

The antimicrobial activity of bimetallic Se-Ag NPs, start materials (sodium selenite and silver nitrate), standard antibiotic Ampicillin-Sulbactam (SAM), and standard antifungal Nystatin (NS) against *Escherichia coli* ATCC25922, *Pseudomonas aeruginosa* ATCC 27853, *Klebsiella oxytoca* ATCC 51983, *Bacillus subtilis* ATCC 6051, *Staphylococcus aureus* ATCC 25922, *Candida albicans* ATCC 90028 was evaluated using the agar well diffusion technique. Agar diffusion test was carried out according to the M51-A2 document of the Clinical Laboratory Standard Institute [80] with modifications. Muller-Hinton agar and Malt extract agar plates were streaked with tested bacterial and fungal strains individually. Using a sterile cork-porer (800), agar wells were made, and then 100 µL of each bimetallic Se-Ag NPs, sodium selenite and silver nitrate standard antibiotic/antifungal at concentrations of 100 µg/mL were put, and incubated at 37 °C for 24 h and 30 °C for 72 h for bacteria and fungi, respectively. After that, the diameter of the inhibition zone of each treatment was measured. To determine the minimum inhibitory concentration, different concentrations of each bimetallic Se-Ag NPs, SAM, and NS were prepared and evaluated for antimicrobial activity [81].

### 3.6. In Vitro Cytotoxicity and Anticancer Activity

The MTT assay was used to evaluate the cytotoxicity of biosynthesized bimetallic Se-Ag NPs toward normal cell line (Wi38) and cancerous cell line (MCF7) [82]. Cell viability and cell inhibition percentages were calculated according to Equations (1) and (2).

(1) $$Viability\;\%=\frac{Test\;OD}{Control\;OD}\times 100$$

(2) Inhibition %=100−Viability %

## 4. Conclusions

A simple and inexpensive method was established for the green and eco-friendly synthesis of bimetallic Se-Ag NPs using WR extract as a reducing and stabilizing agent for the first time. The excellent purity of these bimetallic NPs was confirmed by XRD and TEM/SAED diffracto-grams. After being described, it was found that the average size of bimetallic Se-Ag NPs was 24.5 nm; however, the particle size that DLS measured was 47.3 nm. XRD measurements revealed the bimetallic Se-Ag NPs crystallite size to be 23.84 nm. Different peaks identified by FTIR analysis are related to various groups that were used to stabilize and reduce bimetallic Se-Ag NPs. Results of cytotoxicity confirmed that bimetallic Se-Ag NPs are safe in use, where the IC50 was 168.42 µg/mL toward the Wi38 normal cell line. Furthermore, the results revealed that bimetallic Se-Ag NPs have potential antimicrobial activity against *E. coli*, *P. aeruginosa*, *K. oxytoca*, *B. subtilis*, *S. aureus* and *C. albicans*. Moreover, bimetallic Se-Ag NPs exhibited promising anticancer activity toward the MCF7 cancerous cell line. Therefore, the biosynthesized bimetallic Se-Ag NPs can be used for fighting microbial infections and cancers after in vivo studies. Further research is needed to determine the anticancer and antibacterial mechanisms of the bimetallic Se-Ag NPs in this work in order to optimize their physicochemical characteristics and improve their selectivity.

## Figures and Tables

**Table 1:** Inhibition zones and MICs of biosynthesized bimetallic Se-Ag NPs.

Test Microbial Strain	Se (IZ)	Ag (IZ)	Se-Ag NPs		SAM/NS	
			IZ/mm	MIC (µg/mL)	IZ/mm	MIC (µg/mL)
*E. coli*	ND	ND	14.33 ± 0.58	50	10.33 ± 0.58	100
*P. aeruginosa*	ND	ND	18.87 ± 0.76	12.5	ND	ND
*K. oxytoca*	ND	ND	20.00 ± 1.00	12.5	ND	ND
*B. subtilis*	ND	ND	16.17 ± 0.76	25	12.17 ± 1.04	50
*S. aureus*	ND	ND	17.17 ± 1.26	12.5	ND	ND
*C. albicans*	ND	ND	12.27 ± 1.42	50	ND	ND

Se, Ag, SAM, NS, IZ and ND mean sodium selenite, silver nitrate, Ampicillin-Sulbactam, Nystatin, inhibition zone and not detected.

## Data Availability

The data are made available upon request.
